# Supplementary figures and images for: Mesenchymal stromal cell therapy restores intestinal integrity and attentuates inflammation in a preterm piglet model of necrotizing enterocolitis
Source: Pediatr Surg Int. 2026 Mar 9;42(1):148. doi: 10.1007/s00383-026-06324-7 (PMC12971755; doi:10.1007/s00383-026-06324-7)

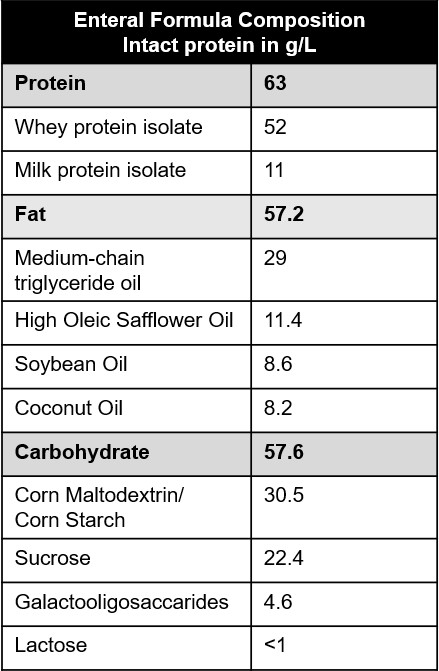

Supplement: Supplementary file 1 — Supplementary Material 1 [file 383_2026_6324_MOESM1_ESM.tiff]

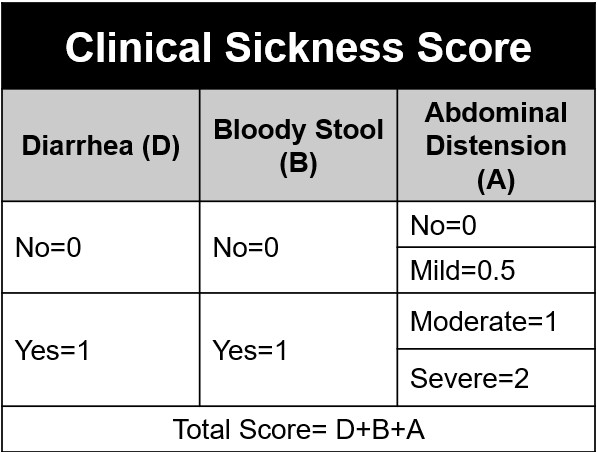

Supplement: Supplementary file 2 — Supplementary Material 2 [file 383_2026_6324_MOESM2_ESM.tif]
